# Human Metapneumovirus Reinfection in Aged Mice Recapitulates Increased Disease Severity in Elderly Humans Infected with Human Metapneumovirus

**DOI:** 10.4049/immunohorizons.2300026

**Published:** 2023-06-01

**Authors:** Olivia B. Parks, Taylor Eddens, Yu Zhang, Tim D. Oury, Anita McElroy, John V. Williams

**Affiliations:** * Department of Pediatrics, Division of Infectious Diseases, University of Pittsburgh School of Medicine, Pittsburgh, PA; † Department of Pediatrics, Division of Allergy/Immunology, University of Pittsburgh School of Medicine, Pittsburgh, PA; ‡ Department of Pathology, University of Pittsburgh School of Medicine, Pittsburgh, PA; § Institute for Infection, Inflammation, and Immunity in Children (i4Kids), Pittsburgh, PA; ¶ Center for Vaccine Research, University of Pittsburgh School of Medicine, Pittsburgh, PA

## Abstract

Human metapneumovirus (HMPV) is a leading cause of respiratory infection in adults >65 y. Nearly all children worldwide are seropositive for HMPV by age 5 y, but reinfections occur throughout life, and there is no licensed vaccine. Recurrent HMPV infection is mild and self-resolving in immunocompetent individuals. However, elderly individuals develop severe respiratory disease on HMPV reinfection that leads to a high risk for morbidity and mortality. In this study, we developed a mouse model to mirror HMPV reinfection in elderly humans. C57BL/6J mice were infected with HMPV at 6–7 wk old, aged in-house, and rechallenged with high-dose virus at 70 wk. Aged rechallenged mice had profound weight loss similar to primary infected mice, increased lung histopathology, and accumulated cytotoxic CD8^+^CD44^+^CD62L^−^CD69^+^CD103^+^ memory cells despite having undetectable lung virus titer. When aged mice 14 mo postinfection (p.i.) or young mice 5 wk p.i. were restimulated with HMPV cognate Ag to mimic epitope vaccination, aged mice had an impaired CD8^+^ memory response. Convalescent serum transfer from young naive or 5 wk p.i. mice into aged mice on day of infection did not protect. Aged mice vaccinated with UV-inactivated HMPV also exhibited diminished protection and poor CD8^+^ memory response compared with young mice. These results suggest aged individuals with HMPV reinfection have a dysregulated CD8^+^ memory T cell response that fails to protect and exacerbates disease. Moreover, aged mice exhibited a poor memory response to either epitope peptide or UV-inactivated vaccination, suggesting that aged CD8^+^ T cell dysfunction presents a barrier to effective vaccination strategies.

## Introduction

Human metapneumovirus (MPV; HMPV) is a leading cause of respiratory infection in children <2 y, adults >65 y, and the immunocompromised (1, 2). Despite children reaching 100% HMPV seroprevalence worldwide by age 5 y, reinfections occur throughout life but usually manifest as mild, self-resolving illness (1, 2). However, HMPV infection in the elderly can result in severe disease with an increased risk for mortality and comorbidities such as bacterial pneumonia (3–6). HMPV prevalence in older adults is similar to influenza and respiratory syncytial virus (RSV), underscoring the burden of these viruses in the aged population (7). The contribution of immune memory response to HMPV in the aged host is not well understood (3, 7–9). One study using primary infection of 18-mo-old BALB/c mice compared with 6- to 7-wk-old mice found that HMPV-infected aged mice had increased weight loss, viral burden, and elevated proinflammatory cytokine production (10). However, memory T cell formation and response to HMPV re-exposure were not assessed. Previous studies have also shown that increased age leads to a progressive decline in function of several immune cell types, which contributes to impaired cell-mediated responses and maintenance of memory T cells leading to poor vaccine responses (11, 12). Thus, impaired immune memory in older adults may contribute to the severity of HMPV reinfection, and it represents a gap in our knowledge.

Aged humans accumulate a population of CD69^+^CD8^+^ tissue-resident memory (T_RM_)-like cells in the lung that is not present in young adults (13). This population of CD69^+^CD8^+^ T_RM_ cells is the main driver of chronic lung sequelae and fibrosis in aged mice infected with influenza virus (14, 15). Moreover, depletion of CD8^+^ T_RM_ cells after primary influenza virus infection in aged mice led to decreased inflammatory monocyte recruitment and diminished lung fibrosis (14), indicating a role for CD8^+^ T_RM_ cells in lung damage after influenza infection.

In addition to altered T cell function, humoral immunity is affected by age, because older adults have a diminished Ab response to inactivated influenza vaccine (16) that is enhanced by adjuvanted and high-dose vaccines (17, 18). HMPV reinfections do occur in older adults despite the presence of humoral immunity (19). Furthermore, reinfection by RSV in humans and mice can occur despite high neutralizing Ab titer (9, 20). These data underscore that humoral immunity alone is not sufficient to provide protection against HMPV in the aged host. We thus sought to define the CD8^+^ T cell memory response in the aged host and elucidate cell-mediated mechanisms of severe HMPV reinfection.

In this study, we established an aged mouse model that mimics reinfection in elderly humans, using mice correlated in age to a human >65 y old (21). Mice 6–7 wk old were infected with HMPV, aged, and rechallenged with high-dose virus 14 mo after primary infection. Aged rechallenged mice had no detectable virus in lungs, yet they exhibited severe weight loss and accumulated CD8^+^CD69^+^CD103^+^ T cells with more potent cytotoxic functions than both aged and young primary infected mice. HMPV epitope vaccination of aged mice 14 mo postinfection (p.i.) proved to be ineffective at inducing a CD8^+^ memory response. Furthermore, vaccination strategies with UV-inactivated virus or HMPV cognate peptide 5 wk before infection with live virus did not improve outcomes in aged mice. Overall, these data show the aged mouse HMPV rechallenge model resembles what is reported in elderly humans. These results also underscore the poor vaccine response in the aged host and show that although immune memory reduced detectable lung virus, it did not protect against disease in aged mice and, in fact, induced immune-mediated lung damage.

## Materials and Methods

### Mice and viral infection

C57BL/6 (B6) mice were purchased from The Jackson Laboratory. All animals were bred and maintained in specific pathogen-free conditions in accordance with the University of Pittsburgh Institutional Animal Care and Use Committee. Female animals 6–7 and 70–71 wk old were used in all experiments. HMPV (pathogenic clinical strain TN/94-49, genotype A2) was grown and titered in LLC-MK2 cells as previously described (22). For all experiments, mice were anesthetized with isoflurane in a heated chamber and infected intratracheally with either 2.0 × 10^6^ or 1.0 × 10^7^ PFUs in 100 μl volume depending on the experiment. Mock-infected mice were inoculated with the same volume of sterile PBS. Viral titers were measured by plaque assay as previously described (22, 23). To generate UV-inactivated HMPV, we placed TN/94-49 virus stock in a sterile six-well tissue culture dish and placed in the Stratagene 1800 ultraviolet Stratalinker run automatic cross-linking (1200 × 100 μJ) for three 10-min intervals, gently agitating the dish in between each dose. UV-inactivated virus was titered via plaque assay to confirm no replicating virus was present.

### CD45.2 i.v. labeling

Mice were administered 4 μg of CD45.2-BUV496 (catalog no. [Cat#] 741092; BD) in 200 μl sterile PBS via tail-vein injection and were euthanized 3 min postinjection as in Goplen et al. (14).

### N11/LPS treatment

Mice were administered 100 μg HMPV N11 peptide (GenScript Peptide Sequence: LSYKHAIL) and 10 μg LPS (Cat# L2360; Sigma) in 200 μl sterile PBS via i.p. injection. Daily weights were taken, and mice were euthanized 5 d posttreatment.

### Histopathologic score

A 10% formalin was injected into a section of the lower left lung lobe and stored in 10% formalin in histology cassettes (B851000WH; Fisher Scientific). Tissue sections were stained with H&E or Picrosirius red stain by the University of Pittsburgh Medical Center Children’s Hospital of Pittsburgh Histology Core, and slides were imaged at ×200 magnification. Scoring criteria for H&E slides per field included: 0, no inflammation; 1, <25% inflammation; 2, 25–50% inflammation; 3, 50–75% inflammation; 4, >75% inflammation. The score for each sample was added and divided by the total number of fields analyzed to generate the histopathologic score.

### IFN-γ ELISPOT assay

ELISPOT assay and analysis were performed as previously described (24, 25). In select experiments, 10 μg of anti–PD-1 (Cat# BE0033-2; BioXCell), 4-1BB (Cat# BE0239; BioXCell), a combination of both, or rat IgG2a isotype control (Cat# BE0089; BioXCell) was added to ELISPOT wells along with HMPV N_11–18_ peptide. Influenza NP366 peptide (GeneScript Peptide Sequence: ASNENMETM) served as control.

### IgG HMPV ELISA

A total of 1 μg of TN/94-49 HMPV in 1× ELISA coating buffer (Cat# 421701; BioLegend) was plated per well overnight at 4°C. The remainder of the ELISA was performed as in Alvarez and Tripp (26).

### Luminex

Lung homogenates were clarified by centrifugation (10,000 × *g* for 10 min) and analyzed via ProcartaPlex Cytokine & Chemokine 36-Plex Panel 1A (Cat# EPX360-26092-901) per manufacturer’s instructions.

### Convalescent serum transfer

Serum was harvested from young mock- or HMPV-infected mice 5 wk p.i. by terminal bleed. A total of 200 μl of serum was passively transferred into mice as described by Elsegeiny et al. (27).

### Flow cytometry staining

#### Single-cell suspension

Mice were euthanized and the right lung harvested. The lung was cut into 2-mm segments using scissors, resuspended in RPMI/10% FBS, and incubated for 1 h at 37°C with DNase and collagenase. After digestion, the lung was filtered through 70-μm filters, spun at 1500 rpm for 5 min, and the pellet was resuspended in 2 ml ACK Lysis Buffer (A10492-01; Life Technologies) for 1 min. A total of 10 ml RPMI/10% FBS was added after ACK Lysis, and cells were spun at 1500 rpm for 5 min. Cells then underwent either tetramer staining or ex vivo peptide stimulation.

#### Tetramer staining

Cells were incubated with 1:2000 dasatinib in 1X PBS/1% FBS (FACS) for 30 min before adding allophycocyanin-conjugated N_11–18_ 1:200 in FACS/dasatinib for 90 min. Cells were then spun down at 1500 rpm for 3 min and washed 1× with FACS buffer.

#### Ex vivo peptide stimulation

A total of 100 μl of cells was added to a flat-bottom 96-well tissue culture plate. The following was added to cells: 100 μl of 200 μM N11 HMPV peptide or NP366 for irrelevant control diluted 1:10 in RPMI/10% FBS, 6 μl CD107a-PE, and 22 μl brefeldin A (Cat. #51-2301KZ; BD)/Monensin (Cat. #2092KZ; BD). In addition, 1:1000 PMA/ionomycin instead of peptide was added to one aliquot of cells for a positive control. Cells were incubated for 5 h at 37°C.

#### For both conditions

After either tetramer staining or peptide stimulation, cells were stained with Live/Dead dye 1:1000 in PBS for 12 min, washed 1× with PBS, and blocked with anti-CD16/32 Fc block (Cat. #70-0161-M001; Tonbo Biosciences) 1:100 in FACS buffer for 10 min. For surface staining, cells were stained with surface Ab 1:100 in BD Brilliant Stain Buffer (Cat. #566349; BD) for 30 min at 4°C. Cells were spun at 1500 rpm for 3 min and washed 1× with FACS buffer.

#### Intracellular cytokine staining

After cells were stained for surface Abs, cells were fixed for 30 min with eBioscience Foxp3/Transcription Factor Staining Buffer Set (00-5523-00; ThermoFisher) at 4°C, spun at 1640 rpm for 3 min, washed 1× with Foxp3 Fix/Perm Buffer, and stained with 6 μl/Ab in Foxp3 Fix/Perm Buffer for 1 h at 4°C. Cells were spun at 1640 rpm for 3 min, washed 1× with FACS buffer, resuspended in FACS buffer, and stored in the dark at 4°C until they were analyzed on the Cytek Aurora multispectral flow cytometer.

#### Intracellular transcription factor staining

For transcription factor staining, cells were fixed for 18 h in Foxp3 Fix/Perm at 4°C. After fix/perm, cells were washed 1× with Foxp3 Fix/Perm Buffer and stained with 2.5 μl Ab in Foxp3 Fix/Perm Buffer for 1 h at 4°C.

After intracellular staining, cells were spun down at 1640 rpm for 3 min, washed 1× with FACS buffer, resuspended in FACS buffer with 100 μl BioLegend Precision Count Beads (Cat. #424902; BioLegend), and run on the Cytek Aurora multispectral flow cytometer. A full list of Abs used in all experiments is shown in Supplemental Table I. Fluorescence minus one controls were used for all inhibitory receptors and transcription factors. For HMPV tetramer staining, influenza NP366-allophycocyanin tetramers were used as irrelevant controls. Any irrelevant tetramer background staining was subtracted from the final tetramer frequency. Unstained cells from each experiment were fixed for 20 min in 2% PFA and used on the flow cytometer to minimize autofluorescence. Data analysis was performed with FlowJo (v10.8.1). Boolean gating in FlowJo was used to assess inhibitory receptor and functional cytokine coexpression. Patterns were visualized using the SPICE program (National Institute of Allergy and Infectious Diseases).

### Statistical analysis

Data analysis was performed using Prism version 9.0 (GraphPad Software). Comparisons between two groups were performed using an unpaired two-tailed Student *t* test or Mann–Whitney as appropriate. Multiple group comparisons were performed using a one-way or two-way ANOVA as appropriate with correction for multiple comparisons (Dunnett test). A *p* value <0.05 was considered significant. Error bars in each graph represent SEM.

### Study approval

All animals were maintained in accordance with *Guide for the Care and Use of Laboratory Animals* (National Institutes of Health publication no. 85-23, Revised 1985) and were handled according to protocols approved by the University of Pittsburgh Subcommittee on Animal Care (Institutional Animal Care and Use Committee).

## Results

### Aged mice rechallenged with virus 14 mo after primary infection exhibit reduced virus in lung yet more severe disease

**FIGURE 1.**
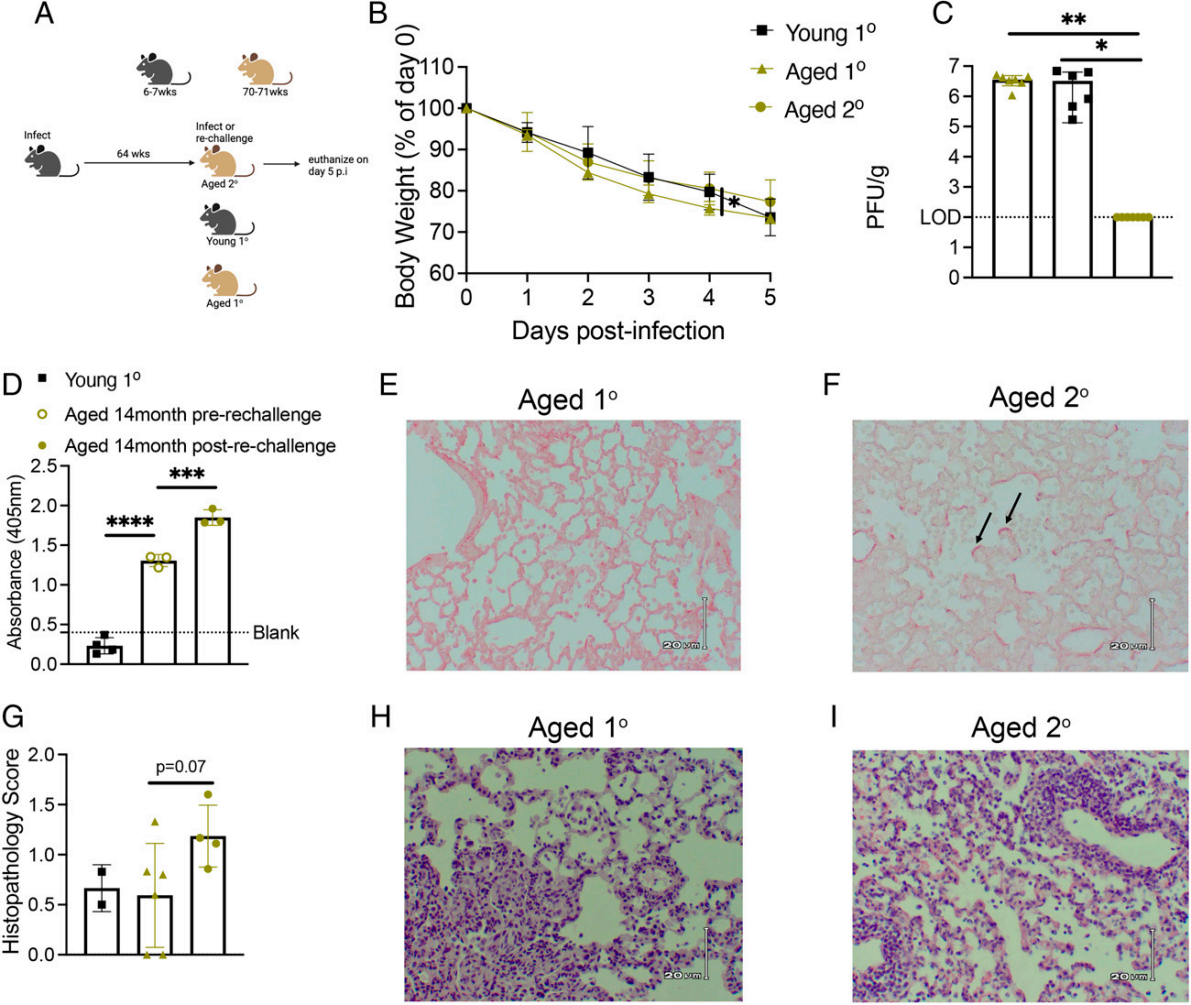
Aged mice rechallenged with virus 14 mo after primary infection exhibit reduced virus in lung yet more severe disease. (**A**) Experimental schematic. (**B**) Aged mice rechallenged with 1 × 10^7^ PFUs had increased weight loss day 5 p.i. similar to aged and young primary infected mice with the same dose. (**C**) Aged rechallenged mice cleared virus by day 5 p.i. (**D**) Aged rechallenged mice had significantly increased HMPV IgG Ab before rechallenge compared with young primary infected mice at day 5 p.i. There was also a significant increase in IgG Ab after rechallenge as measured by absorbance via HMPV-specific ELISA. (**E** and **F**) Aged rechallenged mice have increased linear fibrosis in the alveolar parenchyma denoted by black arrows. (**G**) Aged rechallenged mice tended to have increased pathology scores and inflammation in the lung. (**H** and **I**) Representative histology images. Data in (B) and (C) represent three independent experiments with two to three mice per experiment. Data in (D) and (G) represent one independent experiment. *p < 0.05, **p < 0.01, ***p < 0.005, ****p < 0.0001, one- or two-way ANOVA.

Because most older adults with HMPV are seropositive from prior infection (19), we developed a reinfection model where 6- to 7-wk-old B6 mice were infected with 2 × 10^6^ PFUs of TN/94-49 HMPV, aged in-house for 64 wk, and rechallenged with 1 × 10^7^ PFUs TN/94-49 at 70–71 wk. The TN/94-49 strain at dose 2 × 10^6^ PFUs causes mild, self-resolving disease in young adult B6 mice with minimal weight loss, transient lung inflammation, and viral clearance by days 7–9 p.i., fully protecting against challenge for >8 wk (28). Thus, we chose to rechallenge mice with a higher dose of 1 × 10^7^ PFUs TN/94-49 to overcome immunity from their prior infection (Fig. 1A). Aged mice rechallenged with a higher dose of virus lost weight to a similar degree as aged and young mice receiving primary infection of 1 × 10^7^ PFUs, and all mice were euthanized at day 5 p.i. because of extreme weight loss (Fig. 1B). Despite the severe weight loss similar to primary infected mice, aged rechallenged mice had no detectable titer in lungs at day 5 p.i. (Fig. 1C). In addition, aged mice that were previously infected had a significant HMPV IgG Ab response, which increased further on rechallenge (Fig. 1D). Aged rechallenged mice had increased collagen deposition within the alveolar parenchyma indicative of lung fibrosis compared with aged primary infected mice (Fig. 1E, 1F). Aged rechallenged mice also tended to have more inflammatory infiltrates in lungs (Fig. 1G–I).

### Aged rechallenged mice accumulate CD44^+^CD62L^−^CD69^+^CD103^+^ memory CD8^+^ T cells that have increased cytotoxic function

**FIGURE 2.**
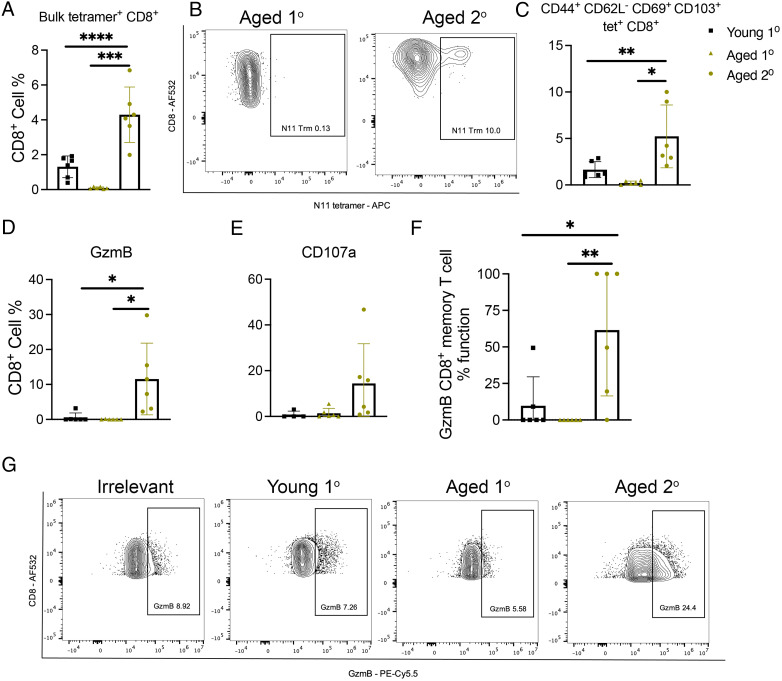
Aged rechallenged mice accumulate CD44^+^CD62L^−^CD69^+^CD103^+^ memory CD8^+^ T cells that have increased cytotoxic function. (**A**) Aged rechallenged mice produced more, but aged primary infected mice produced significantly fewer, tet^+^CD8^+^ T cells compared with young primary infected mice. (**B** and **C**) Aged rechallenged mice accumulated significantly more virus-specific CD44^+^CD62L^−^CD69^+^CD103^+^CD8^+^ T cells in lung compared with aged and young primary infected mice. (**D** and **E**) On ex vivo peptide restimulation, aged rechallenged mice produced significantly more granzyme B and tended to degranulate more as measured by CD107a staining. (**F**) Significantly more of the CD44^+^CD62L^−^CD69^+^CD103^+^CD8^+^ T cells were functional in producing granzyme in the aged rechallenged mice. This was calculated by granzyme B cells percent divided by memory CD8^+^ T cell percent. (**G**) Representative flow plots of granzyme B staining. Data represent three independent experiments with five to six mice per experiment. *p < 0.05, **p < 0.01, ***p < 0.005, ****p < 0.0001, one-way ANOVA.

Aged mice produced fewer tetramer-positive (tet^+^) CD8^+^ T cells after primary HMPV infection compared with young primary infected mice (Fig. 2A; Supplemental Fig. 1A). However, aged mice rechallenged 14 mo after primary infection had increased tet^+^CD8^+^ T cells compared with primary infection in both age groups (Fig. 2A; Supplemental Fig. 1A). Aged rechallenged mice had significantly more CD44^+^CD62L^−^ effector CD8^+^ T cells and significantly fewer CD44^−^CD62L^+^CD8^+^ T cells compared with young mice (Supplemental Fig. 1B, 1C). Aged rechallenged mice accumulated significantly more tet^+^ memory CD8^+^ T cells as defined by CD44^+^CD62L^−^CD69^+^CD103^+^ expression (Fig. 2B, 2C; Supplemental Fig. 1D). On ex vivo peptide restimulation, CD8^+^ T cells from aged rechallenged mice produced significantly more granzyme B (Fig. 2D, 2G) and tended to degranulate more as measured by CD107a (Fig. 2E). In addition, memory CD8^+^ T cells in aged rechallenged mice were more functional, having increased granzyme B production (Fig. 2F). There was no difference in percent or absolute number of CD19^+^ B or CD4^+^ T cells between groups (Supplemental Fig. 1E, 1F). Gating strategies are shown in Supplemental Fig. 2. In addition, we found increased concentration of T cell chemoattractants, including CCL2, CCL3, CCL7, and CXCL2, in the lungs of aged, primary infected mice late in HMPV infection, which could explain the accumulation of CD8^+^ T cells in the lung of aged mice (Supplemental Fig. 3A, 3B). Taken together, aged rechallenged mice exhibited severe disease and lung inflammation despite undetectable virus in lung. Furthermore, aged rechallenged mice accumulated significantly more tet^+^ memory CD8^+^ T cells with increased cytotoxic function, suggesting immune-mediated disease.

### Serum from young mice 5 wk p.i. did not protect aged or young mice against HMPV infection

**FIGURE 3.**
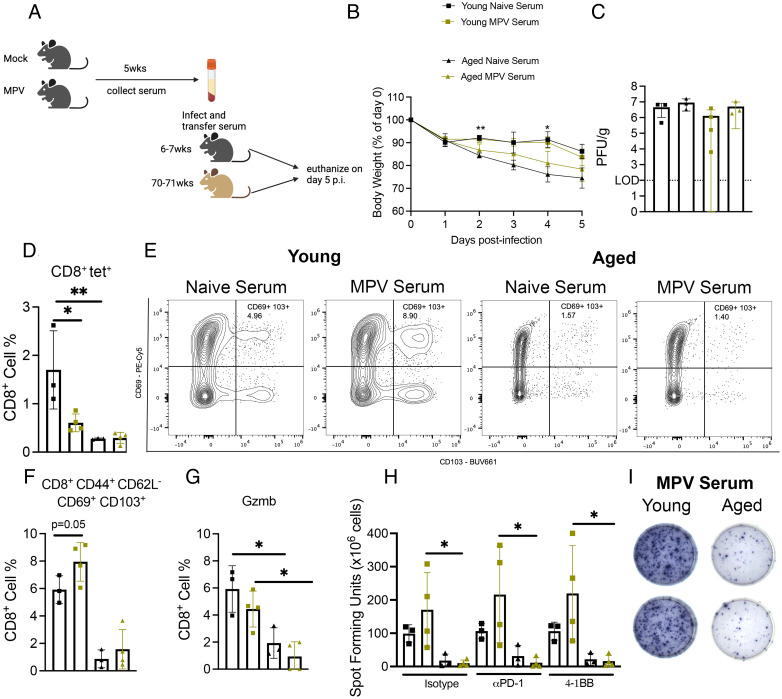
Serum from young mice 5 wk p.i. did not protect aged or young mice against HMPV infection. (**A**) Schematic of experimental design. (**B**) Aged mice that received naive serum lost more weight at days 2 and 4 p.i. (**C**) There was no difference in viral titer between any of the age groups. (**D**) Aged mice produced fewer CD8^+^tet^+^ T cells regardless of whether they received naive or MPV serum. (**E** and **F**) Aged mice also produced fewer CD44^+^CD62L^−^CD69^+^CD103^+^CD8^+^ memory T cells regardless of serum treatment. (**G**) Aged CD8^+^ T cells from both groups produced significantly less granzyme B compared with young mice. (**H** and **I**) Aged CD8^+^ T cells also produced less IFN as measured by ELISPOT. Data represent one independent experiment with three to four mice per group. *p < 0.05, **p < 0.01, one-way ANOVA.

Considering that aged mice rechallenged with virus 14 mo after primary infection had undetectable lung viral titer, we questioned whether the humoral response neutralized the virus on reinfection to abort replication. To test the role of the humoral response during HMPV infection, we collected serum from either naive or HMPV-infected young mice 5 wk p.i. Naive or HMPV serum was injected into aged or young mice on the day of infection (Fig. 3A). Aged mice that received naive serum lost significantly more weight on days 3 and 4 p.i. compared with young mice with naive serum (Fig. 3B). There was no difference in viral titer between all four groups (Fig. 3C). Aged mice that received either naive or HMPV serum produced fewer tet^+^ CD8^+^ T cells compared with young mice (Fig. 3D), although young mice injected with HMPV serum had significantly fewer tet^+^CD8^+^ cells compared with young mice injected with naive serum (Fig. 3D). Aged mice produced fewer CD44^+^CD62L^−^CD69^+^CD103^+^CD8^+^ T cells in the lung regardless of serum given (Fig. 3E, 3F). Young mice that received HMPV serum did have a modest increase in memory CD8^+^ T cells compared with young mice with naive serum (Fig. 3E, 3F). Aged CD8^+^ T cells in both groups produced less granzyme B via ex vivo peptide stimulation (Fig. 3G). In addition, aged T cells stimulated with class I peptide in ELISPOT produced less IFN regardless of serum type (Fig. 3H, 3I). Young mice did tend to produce more IFN when given HMPV serum compared with aged mice with HMPV serum (Fig. 3H, 3I). In an attempt to improve the aged CD8^+^ T cell IFN response, PD-1 blockade or 4-1BB costimulation was added to the ex vivo peptide stimulation, both of which have been used to potentiate CD8^+^ T cells in cancer models (29–31). However, neither treatment increased IFN production in aged CD8^+^ T cells (Fig. 3H, 3I). Taken together, these data suggest that the humoral response is only partially protective in young mice but suggests that the weight loss in aged mice is not mediated by HMPV-specific Ab. These data would support the idea that cellular immunity, such as dysfunctional CD8^+^ T cells, is a critical driver of immunopathology rather than humoral immunity.

### Aged mice failed to contract CD45.2^−^CD8^+^ T cells in the lung 40 d p.i.

**FIGURE 4.**
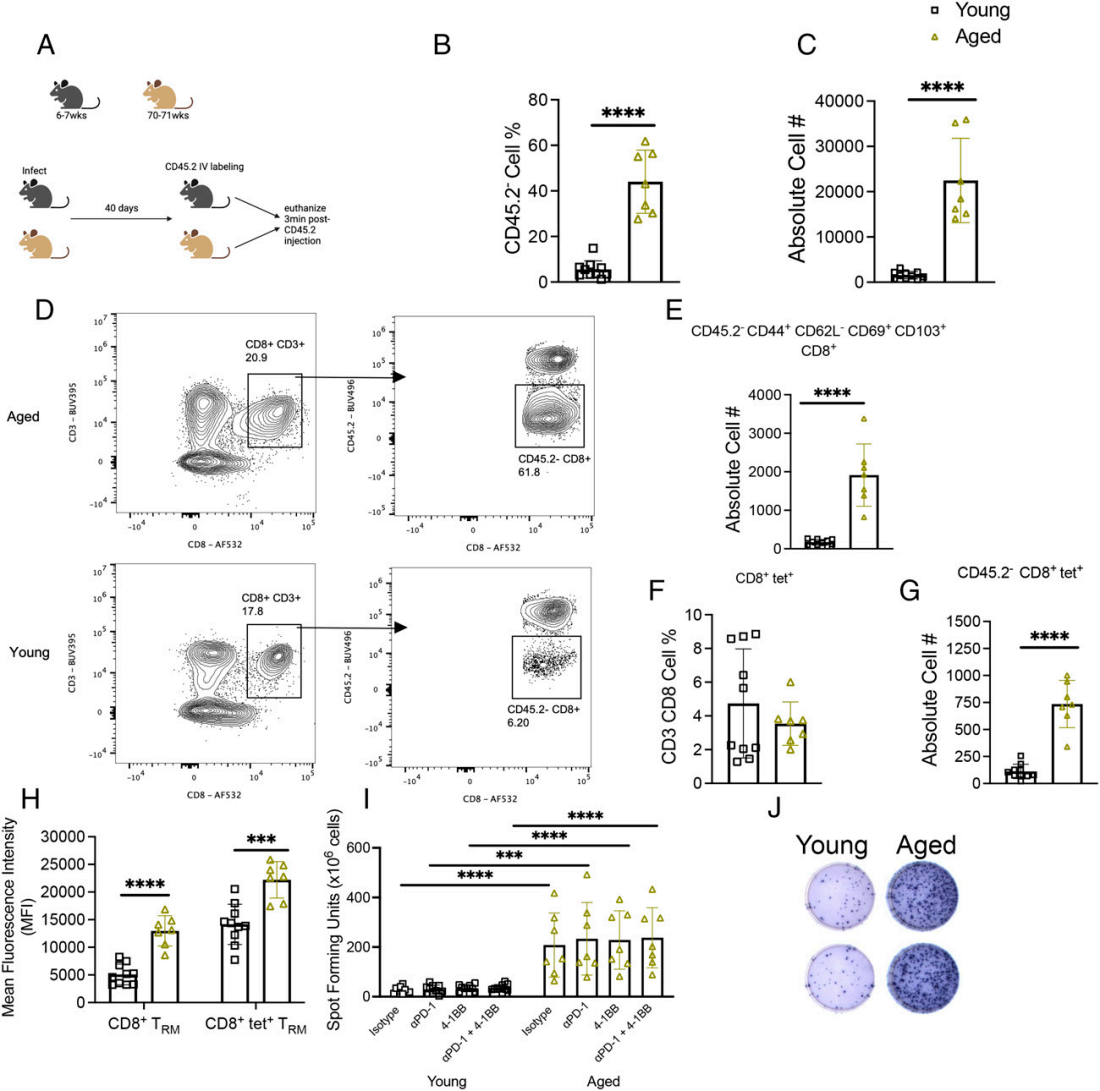
Aged mice failed to contract CD45.2^−^CD8^+^ T cells in the lung 40 d p.i. (**A**) Experimental design schematic. (**B** and **C**) Aged mice had significantly more CD45.2^−^CD8^+^ T cells in the lung via CD45.2 i.v. labeling at day 40 p.i. compared with young mice. (**D**) Representative gating of CD45.2 staining. (**E**) Aged mice had increased absolute number of CD45.2^−^CD44^+^CD62L^−^CD69^+^CD103^+^CD8^+^ (i.e., T_RM_) T cells. (**F**) There was no difference in CD8^+^tet^+^ frequency between age groups. (**G**) Aged mice had increased absolute number of CD45.2^−^CD8^+^tet^+^ T cells in lung. (**H**) PD-1 mean fluorescence intensity expression was increased on aged bulk CD8^+^ and tet^+^ T_RM_ cells in the lung. (**I** and **J**) Aged lung T lymphocytes had significant IFN production after ex vivo peptide stimulation. Data represent two independent experiments with three to five mice per experiment. ***p < 0.005, ****p < 0.0001, unpaired *t* test or one-way ANOVA.

We then sought to compare differences in T cell memory formation between young and aged mice and identify lung tissue-resident CD8^+^ T cells more precisely. Aged and young mice were infected with 1 × 10^6^ PFUs HMPV and 40 d p.i. were injected i.v. with CD45.2 Ab and immediately euthanized (Fig. 4A). Using this in vivo labeling method, tissue-resident cells will stain negative for CD45.2, whereas cells trafficking to the lung from the blood will stain positive (32). Aged mice accumulated significantly more CD45.2^−^CD8^+^ T lymphocytes, consistent with T_RM_ cells, in the lung (Fig. 4B–D). There was a trend toward an increase in frequency of CD45.2^−^CD44^+^CD62L^−^CD69^+^CD103^+^ T_RM_ cells in young mice (Supplemental Fig. 3C). However, by absolute cell number, aged mice accumulated more of these CD8^+^ T_RM_ cells in the lung (Fig. 4E). Although there was no significant difference in the frequency or absolute number of bulk tet^+^CD8^+^ T cells between the age groups (Fig. 4F, Supplemental Fig. 3D), aged mice produced more CD45.2^−^tet^+^ CD8^+^ T cells (Fig. 4G). Both bulk and tet^+^ aged CD45.2^−^CD8^+^ T had increased mean fluorescence intensity and cell percent expressing PD-1 compared with young mice (Fig. 4H, Supplemental Fig. 3E). This led us to consider whether treating lung lymphocytes with PD-1 blockade and 4-1BB stimulation would impact CD8^+^ T cell function in this model. Ex vivo peptide restimulation of lung lymphocytes revealed that aged mice exhibited significantly more IFN-γ–producing cells compared with young mice (Fig. 4I, 4J). However, PD-1 blockade, 4-1BB agonist treatment, or the combination did not change IFN-γ production in either age group (Fig. 4I). There was no difference in HMPV IgG production between aged and young mice (Supplemental Fig. 3F). These results suggest that aged mice accumulate CD45.2^−^CD8^+^ T_RM_ cells in the lung after primary infection, and that these aged CD8^+^ T cells have increased cytotoxic function that is not restrained by PD-1 or 4-1BB signaling.

### Aged mice restimulated with HMPV peptide/LPS adjuvant lost more weight and had diminished CD8^+^ memory response

**FIGURE 5.**
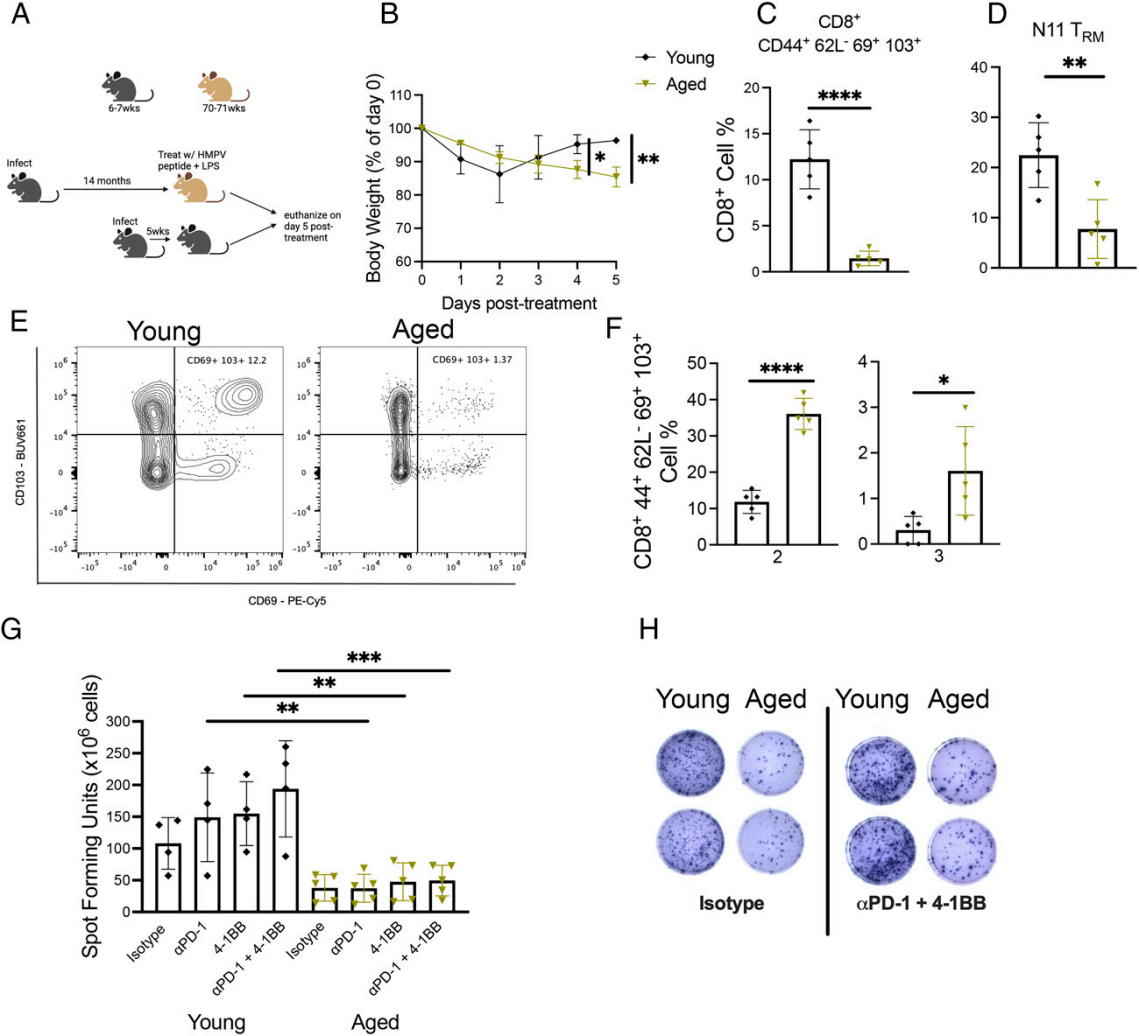
Aged mice restimulated with HMPV-peptide/LPS adjuvant lost more weight and had diminished CD8^+^ memory response. (**A**) Experimental design. (**B**) Aged mice restimulated with HMPV N11 peptide + LPS lost significantly more weight posttreatment. (**C**–**E**) Aged restimulated mice had diminished bulk and virus-specific CD8^+^ T_RM_ response. (**F**) CD8^+^CD44^+^CD62L^−^CD69^+^CD103^+^ T cells from aged restimulated mice coexpressed significantly more inhibitory receptors PD-1, TIM-3, LAG-3, and 2B4 based on combinatorial analysis. (**G** and **H**) Aged restimulated mice produced less IFN at baseline in ex vivo peptide stimulation and failed to increase IFN production with PD-1, 4-1BB, or combination therapy. Data represent one experiment with five mice per group. *p < 0.05, **p < 0.01, ***p < 0.005, ****p < 0.0001, unpaired *t* test.

Elderly humans have a poor response to vaccines (33–35). We aimed to use this aged mouse model to elucidate the CD8^+^ T cell memory response after vaccinating aged mice 14 mo p.i. or young mice 5 wk p.i. with HMPV cognate peptide Ag for a dominant K^b^/N_11–19_ (N11) epitope (24, 28) adjuvanted with LPS via i.p. injection (Fig. 5A). This model was meant to mimic what would occur if elderly humans received epitope or inactivated vaccination for a virus they had previously been exposed to. Aged mice lost significantly more weight compared with young mice (Fig. 5B) and accumulated fewer bulk and tet^+^ memory CD8^+^ T cells in the lung (Fig. 5C–E, Supplemental Fig. 4A). Aged mice also produced fewer tet^+^CD8^+^ T cells compared with young mice (Supplemental Fig. 4B), as we observed in aged mouse primary infection (Fig. 2A). There was no difference in CD8^+^ absolute cell number in the lung between the age groups (Supplemental Fig. 4C), which ruled out a global impairment in aged CD8^+^ T cell production. Because aged mice exhibited a diminished CD8^+^ memory response to peptide stimulation in this model, we hypothesized that HMPV cognate Ag and LPS adjuvant did not adequately activate the CD8^+^ T cell response in aged mice. To test this hypothesis, we assessed inhibitory receptor expression on CD8^+^ memory T cells. Combinatorial analysis of PD-1, TIM-3, LAG-3, and 2B4 expression on bulk CD8^+^ memory T cells revealed that the majority of memory CD8 T cells from young mice had increased expression of only one inhibitory receptor (Supplemental Fig. 4D), whereas aged mice coexpressed two or three of these receptors (Fig. 5F), but neither age group expressed all four (Supplemental Fig. 4E). Analysis of CD44 and CD62L expression on CD8^+^ T cells showed that aged mice had increased CD44^+^CD62L^−^CD8^+^ T effector cells and a significantly diminished pool of CD44^−^CD62L^+^CD8^+^ T cells (Supplemental Fig. 4F). Furthermore, ex vivo HMPV peptide stimulation ELISPOT revealed that aged mice generated significantly fewer IFN-γ–producing cells compared with young mice whether treated with PD-1 blockade, 4-1BB agonist treatment, or both (Fig. 5G, 5H). These results indicate that aged mice mounted an impaired CD8^+^ memory response when rechallenged with HMPV cognate peptide Ag in the absence of viral infection.

**FIGURE 6.**
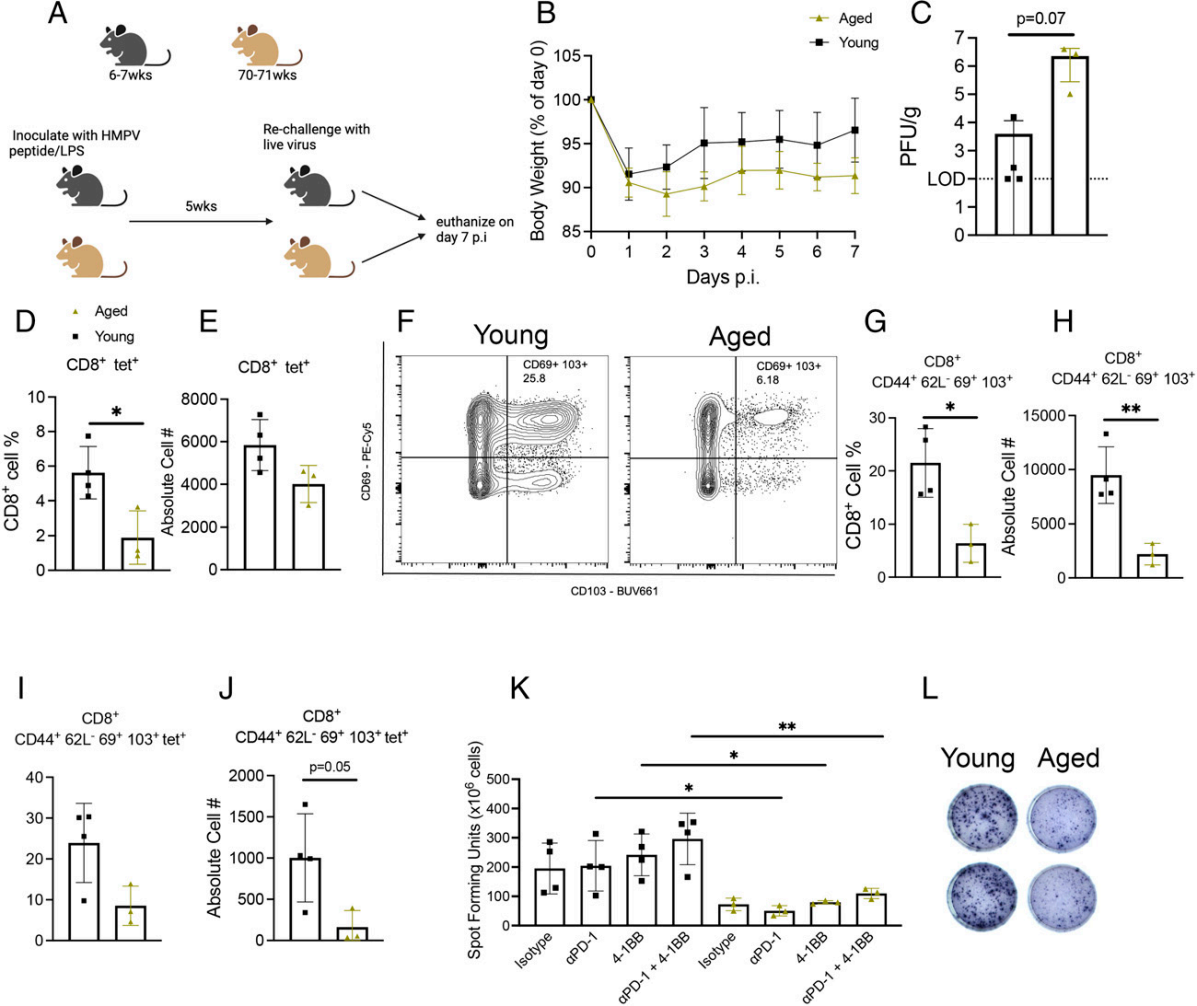
Aged mice vaccinated with HMPV cognate peptide before infection had poor CD8^+^ memory response. (**A**) Schematic of experimental design. (**B**) Aged vaccinated mice tended to lose more weight p.i. (**C**) Aged vaccinated mice also tended to have higher titer in lung. (**D** and **E**) Aged mice produced fewer tet^+^CD8^+^ T cells. (**F**–**H**) Aged vaccinated mice also produced fewer CD8^+^CD44^+^CD62L^−^CD69^+^CD103^+^ memory T cells compared with young mice. (**I** and **J**) Aged vaccinated mice tended to also have fewer tet^+^ memory CD8^+^ T cells. (**K** and **L**) Aged CD8^+^ T cells also produced less IFN as measured by ex vivo peptide stimulation ELISPOT. Data represent one independent experiment with three to four mice per group. *p < 0.05, **p < 0.01, unpaired *t* test.

To further test this vaccine strategy, we vaccinated aged and young mice with HMPV cognate peptide/LPS, waited 5 wk, then rechallenged with live virus and assessed the CD8^+^ memory response (Fig. 6A). Aged mice tended to lose more weight p.i. (Fig. 6B) and have more virus in lungs (Fig. 6C). Aged mice produced fewer tet^+^CD8^+^ T cells (Fig. 6D, 6E), as well as CD44^+^CD62L^−^CD69^+^CD103^+^CD8^+^ memory T cells (Fig. 6F–H) and tet^+^CD8^+^ memory T cells (Fig. 6I, 6J). In addition, aged vaccinated mice produced less IFN via ex vivo peptide stimulation, which was not improved with PD-1 blockade or 4-1BB costimulation (Fig. 6K, 6L). Taken together, these results indicate that HMPV cognate peptide/LPS is an ineffective vaccine in aged mice.

### Aged mice vaccinated with UV-inactivated HMPV exhibited severe HMPV disease and poor CD8^+^ memory response

**FIGURE 7.**
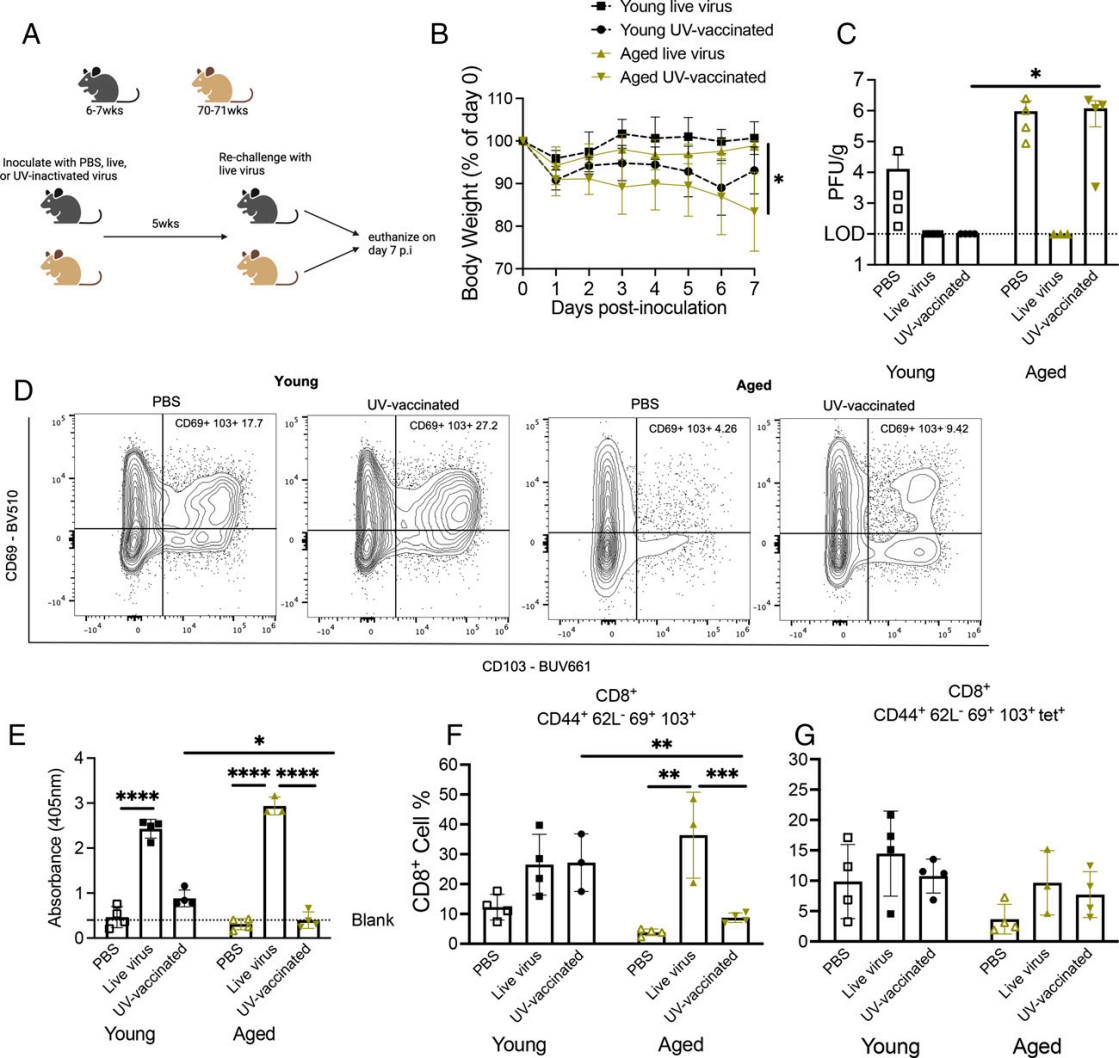
Aged mice vaccinated with UV-inactivated HMPV exhibited severe HMPV disease and poor CD8^+^ memory response. (**A**) Schematic of experimental design. (**B**) Aged mice primed with UV-inactivated virus and rechallenged with live virus lost significantly more weight p.i. compared with aged mice primed with live virus. (**C**) Aged mice primed with UV-inactivated virus had significantly higher viral titer at day 7 p.i. (**D**) Aged and young mice primed with live virus mounted a robust IgG Ab response compared with UV-inactivated virus priming. (**E** and **F**) Aged mice primed with UV-inactivated virus had significantly decreased CD8^+^CD44^+^CD62L^−^CD69^+^CD103^+^ cell percent compared with aged mice primed with live virus or young mice primed with UV-inactivated virus. There was no significant difference in virus-specific memory CD8^+^ T cells. (**G**) Representative flow plots of CD69 and CD103 staining. Data represent two independent experiments with two to three mice per group. *p < 0.05, **p < 0.01, ***p < 0.005, ****p < 0.0001, one-way ANOVA.

We have shown that rechallenged aged mice develop severe disease and lung inflammation while accumulating dysfunctional CD8^+^ T_RM_ cells. Conversely, nonreplicating peptide + LPS injection given as vaccination before or after HMPV infection did not stimulate an adequate CD8^+^ T cell response in aged mice. Because vaccination of older individuals is a highly desirable preventive strategy, we next aimed to assess the aged CD8^+^ memory response in another vaccination model. Aged 70- to 71-wk-old or young 6- to 7-wk-old B6 mice were primed with either PBS, 2 × 10^6^ PFU live virus, or an equivalent volume of UV-inactivated virus followed by rechallenge 5 wk later with live virus (Fig. 7A). Aged mice primed with UV-inactivated virus lost significantly more weight after rechallenge compared with aged mice primed with live virus (Fig. 7B). Aged mice primed with PBS (thus undergoing primary infection) tended to have higher viral burden in lung compared with young primary infected mice (Fig. 7C), a phenomenon we have previously observed (O.B. Parks, T. Eddens, J. Sojati, J. Lan, Y. Zhang, T.D. Oury, M. Ramsey, J.J. Erickson, C.A. Byersdorfer, and J.V. Williams, submitted for publication). Aged mice vaccinated with UV-inactivated virus had significantly higher viral titer compared with young UV-vaccinated mice, which lacked detectable lung virus (Fig. 7C). Aged and young mice primed with live virus had undetectable titer in lungs (Fig. 7C). IgG HMPV serum ELISA showed a robust IgG response by both aged and young mice primed with live virus (Fig. 7E). However, aged UV-vaccinated mice had negligible IgG response (Fig. 7E). Young UV-vaccinated mice had significantly more IgG response compared with aged UV-vaccinated mice but still less than young mice primed with live virus (Fig. 7E).

We next assessed the lung CD8^+^ memory T cell response to this vaccination model. Aged UV-vaccinated mice had a significantly decreased bulk CD8^+^ memory T cell response compared with young UV-vaccinated mice, whereas mice of both age groups primed with live virus had a robust bulk CD8^+^ memory T cell response (Fig. 7D, 7F). There was also a modest, but not significant, increase in tet^+^CD8^+^ memory T cells in young mice primed with live virus compared with aged mice (Fig. 7G). Representative flow plots of CD69^+^CD103^+^ staining are shown in Fig. 7D. Because we observed this difference in CD8^+^ memory T cells between the age groups and based on vaccination or live virus priming, we performed combinatorial analysis to assess PD-1, TIM-3, LAG-3, and 2B4 inhibitory receptor coexpression on CD8^+^ memory T cells. Aged and young vaccinated mice had significantly increased coexpression of three inhibitory receptors compared with aged and young mice primed with live virus with no significant difference in zero, one, two, or four inhibitory coexpressions (Supplemental Fig. 4G, 4H). Taken together, these data indicate that vaccinating aged mice with UV-inactivated virus led to less effective IgG Ab production and more impaired CD8^+^ memory T cells, resulting in increased weight loss and delayed viral clearance.

## Discussion

In this study, we developed an HMPV rechallenge aged mouse model to recapitulate human disease, where initial exposure occurs in childhood and re-exposure occurs again later in life. We found that aged rechallenged mice developed severe disease comparable with primary infected aged and young mice despite undetectable lung virus titer. Aged rechallenged mice had a robust Ab response, suggesting this protected against lung virus replication. Nonetheless, aged rechallenged mice exhibited more severe disease, lung inflammation, and fibrosis and increased cytotoxic CD8^+^ T cell response, all suggesting immune-mediated pathology. Aged rechallenged mice did exhibit a greater number of lung CD8^+^ T_RM_ cells, but these cells demonstrated enhanced effector functions. We also observed an increase in chemoattractants in the aged lung late in HMPV infection (i.e., days 7–9), which could explain, in part, the accumulation of lung CD8^+^ T cells in aged mice. We further show that the humoral response is not fully protective against HMPV infection because even young mice given serum from young mice 5 wk after MPV infection had only a small decrease in lung viral titer.

We previously showed that PD-1 signaling restrains antiviral CD8^+^ T cell functions in young adult mice (24, 28, 36, 37). However, aged reinfected mice CD8^+^ T cells demonstrated strong effector function in the presence or absence of PD-1 blockade. Similarly, 4-1BB agonists restore function to exhausted T cells in some cancer models (29–31), but blocking this pathway had no effect on the aged antiviral CD8^+^ T cells. Importantly, there was also no effect of PD-1 blockade or 4-1BB agonist in young mice at day 40 p.i. One explanation for these findings in young mice could be that young mice contracted their memory CD8^+^ T cell population in the lung by day 40 p.i., minimizing any differences that these agents would produce. When young mice were restimulated with HMPV peptide/LPS adjuvant, causing the memory CD8^+^ T cell population to expand, they exhibited a trend toward increased IFN-γ production with PD-1/4-1BB combination, supporting our previous findings of PD-1 signaling in young mice (24, 28, 36, 37). In contrast, aged mice failed to respond to PD-1 blockade of 4-1BB agonist treatment in either model. Collectively, these data suggest that older mice developed a strong but ultimately dysfunctional and perhaps unregulated memory CD8^+^ T cell response. Similar to our findings, in aged mice infected with influenza, cytotoxic CD8^+^CD69^+^ T_RM_ cells accumulated up to 60 d postprimary infection and caused significant lung inflammation and fibrosis (14). These findings suggest that HMPV severe respiratory disease in the elderly could, in part, be driven by these cytotoxic dysfunctional CD8^+^ memory T cells.

To test HMPV vaccination strategies, we rechallenged aged and young previously infected mice with HMPV cognate peptide Ag 14 mo or 5 wk after primary infection. We hypothesized that a nonreplicating immune memory stimulus with HMPV peptide plus LPS would result in an improved immune response in aged rechallenged mice and induce a less cytotoxic T cell response. However, aged mice restimulated with HMPV cognate Ag mounted a poor CD8^+^ T_RM_ response that coexpressed more inhibitory receptors and produced less IFN-γ. In our complementary vaccination approach, aged mice primed with HMPV cognate peptide plus LPS also had a poor memory response on rechallenge with live virus. In contrast, the robust accumulation of CD8^+^ memory T cells with low inhibitory receptor expression we observed in young mice is similar to our previous vaccination strategies using virus-like particles (38) and dendritic cell vaccination (36). LPS alters the immune response in female mice that are 12 mo old (39), so it is possible that using LPS as an adjuvant was detrimental to the immune response in aged mice leading to an impaired CD8^+^ memory response and weight loss. These findings in our HMPV peptide restimulated aged mice may suggest why elderly humas exhibit a poor response to vaccines against viruses to which they have previously been exposed. Overall, these data suggest an intrinsic defect in the aged CD8^+^ memory T cell population that causes immunopathology on re-exposure to either live virus or HMPV peptide vaccination later in life.

Older adults mount a poor memory response to many vaccines, and adjuvanted or high-dose vaccines are often required (16–18). Currently, there is no licensed HMPV vaccine, although we and others have tested vaccine strategies in rodents (36, 38, 40), nonhuman primates (41, 42), and humans (43, 44). UV-inactivated vaccines have been tested in mice for other respiratory viruses and elicited promising results (45, 46). Therefore, we tested whether a UV-inactivated vaccine approach might elicit robust T cell–mediated immunity in aged mice. Aged UV-vaccinated mice exhibited severe disease with increased weight loss and delayed viral clearance compared with young UV-vaccinated mice. In addition, aged vaccinated mice had a lower IgG Ab response and a diminished CD8^+^ memory T cell response with increased coexpression of inhibitory receptors. Thus, vaccinating aged mice with UV-inactivated HMPV was inadequate to protect against exposure to live HMPV. These findings underscore the dysfunctional CD8^+^ memory T cell response in the aged host and emphasize the importance of optimizing respiratory virus vaccine strategies in this vulnerable population. One recent study showed improved protection against RSV after elderly humans were vaccinated with AS01_E_-adjuvanted RSV F protein–based vaccine (47). These findings introduce possible HMPV vaccine strategies to pursue in future studies with our aged mouse model. Future studies are warranted to elucidate the mechanism behind the poor memory response to a UV-inactivated vaccine in aged mice.

Taken together, to our knowledge, we developed a novel 14-mo rechallenge mouse model that resembles viral reinfections in elderly humans. We demonstrate multiple ways to use this model to elucidate the aged immune response to rechallenge. This aged mouse model of HMPV reinfection also provides insight into CD8^+^ memory T cell contribution to severe HMPV disease in aged rechallenged mice. Furthermore, this model will be useful to optimize vaccine development in this vulnerable population.

## Supplementary Material

Supplemental Figures 1 (PDF)
