# Supplementary material for: Human Metapneumovirus Reinfection in Aged Mice Recapitulates Increased Disease Severity in Elderly Humans Infected with Human Metapneumovirus
Source: Immunohorizons. 2023 Jun 1;7(6):398–411. doi: 10.4049/immunohorizons.2300026 (PMC10321313; doi:10.4049/immunohorizons.2300026)
Supplement: Supplemental Figures 1 (PDF) [file IH_2300026_Supplemental_1.pdf]

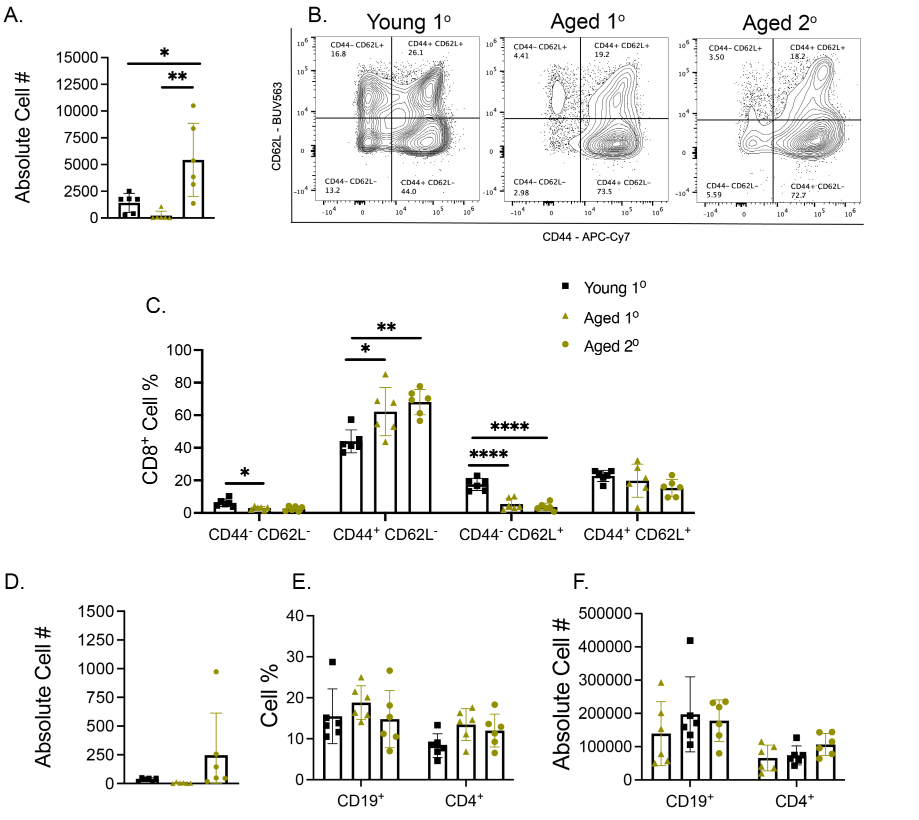

Supplemental Figure 1. Aged re-challenged mice have fewer naive $CD8^+$ $CD44^-$ $CD62L^+$ T cells but no difference in $CD19^+$ or $CD4^+$ T cells.

(A) Aged re-challenged mice had increased absolute cell number of tet^+ $CD8^+$ T cells. **(C)** Representative flow plots showing CD44 and CD62L expression on $CD8^+$ T cells. **(C)** Aged re-challenged mice had fewer $CD8^+$ $CD44^-$ $CD62L^+$ T cells and increased $CD8^+$ T cells expressing $CD44^+$ $CD62L^-$. **(D)** Aged re-challenged had a modest increase in absolute cell number of tet^+ $CD8^+$ memory T cells. **(E-F)** There was no difference in cell percent or absolute cell number of $CD19^+$ or $CD4^+$ cells between the groups. Data represent 3 independent experiments with 5-6 mice per experiment. *P<0.05, **P<0.01, ****P<0.0001, one-way ANOVA.

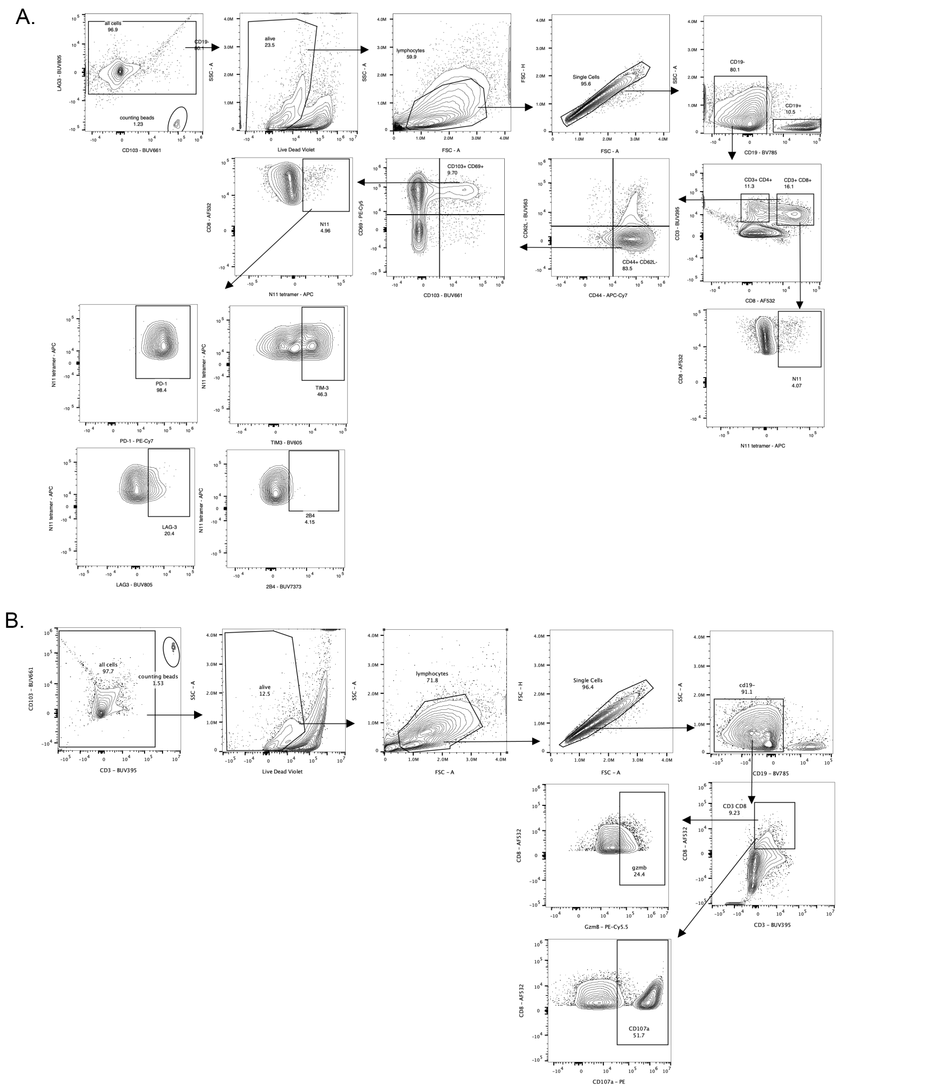

Supplemental Figure 2. CD8⁺ memory T cell and *ex vivo* peptide stimulation gating strategies. **(A)** CD8⁺ memory T cell gating strategy. **(B)** *ex vivo* peptide stimulation gating strategy.

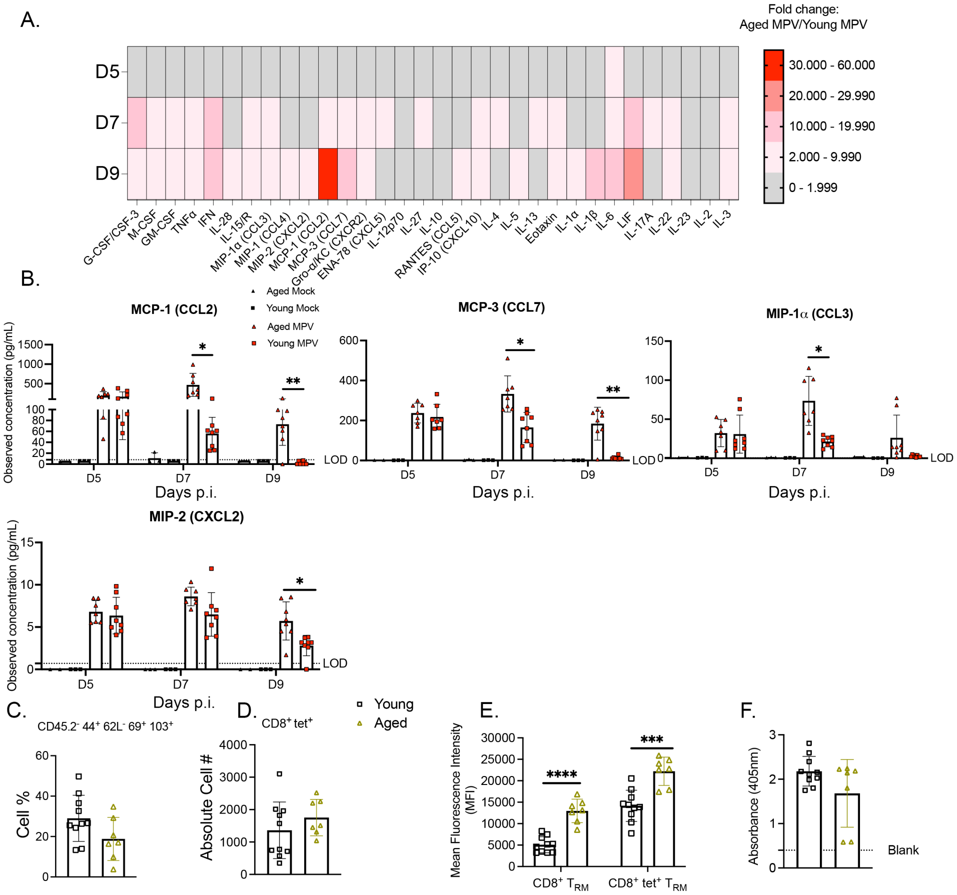

Supplemental Figure 3. Aged mice had increased T cell chemoattractants in lung late during HMPV infection and had increased PD-1 expression on CD45.2⁻ CD8⁺ T cells in the lung 40 days p.i.

(A) Fold change aged MPV/young MPV heat map of chemokine and cytokine expression from lung homogenates. **(B)** Raw data from select chemokines. **(C)** There was no difference between age groups in cell percent of $CD45.2^{-} 44^{+} 62L^{-} 69^{+} 103^{+} CD8^{+}$ TRM cells. **(D)** There was also no difference in $CD8^{+} tet^{+}$ absolute cell number. **(E)** Aged bulk $CD8^{+}$ and $CD8^{+} tet^{+}$ TRMs had increased cell percent PD-1 expression. **(F)** There was no difference in HMPV IgG production as measured by HMPV ELISA. Data represent 2 independent experiments with 3-5 mice per experiment. *P<0.05, **P<0.01,***P<0.005, unpaired t-test or two-way ANOVA

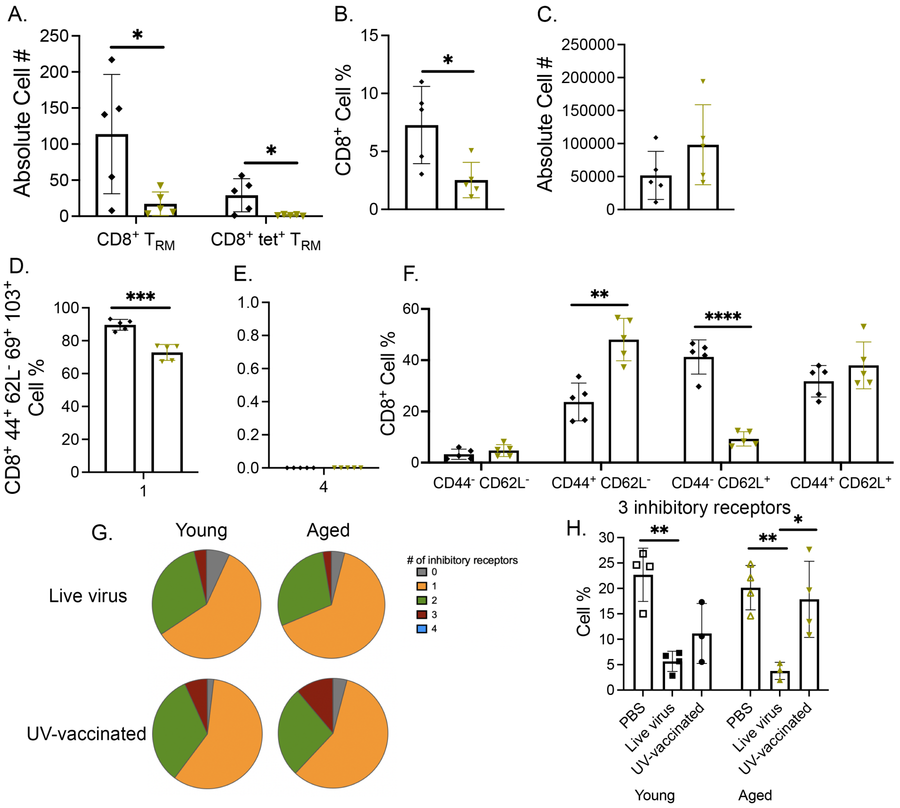

Supplemental Figure 4. Aged mice re-stimulated with HMPV cognate peptide adjuvanted with LPS or vaccinated with UV-inactivated virus had impaired $CD8^+$ memory T cell response. **(A)** Aged mice accumulated fewer bulk and tet^+ $CD8^+$ TRMs. **(B)** Aged mice also produced fewer tet^+ $CD8^+$ T cells compared to young mice. **(C)** There was no difference in $CD8^+$ absolute cell number between the age groups. **(D)** Young mice had increased expression of only one inhibitory receptor on $CD8^+$ TRMs. **(E)** Neither age group expressed all four inhibitory receptors (i.e. PD-1, TIM-3, LAG-3, and 2B4) on $CD8^+$ TRMs. **(F)** Aged mice had increased $CD8^+$ $CD44^+$ $CD62L^-$ T cells but had a smaller pool of naive $CD44^-$ $CD62L^+$ $CD8^+$ T cells compared to young mice. (G) SPICE pie charts of combinatorial analysis of Boolean gating of 0, 1, 2, 3, or 4 inhibitory receptor expression. Inhibitory receptors assessed: PD-1, TIM-3, LAG-3, 2B4. Aged and young vaccinated mice had increased co-expression of 3 inhibitory receptors. **A-F** data represents 1 independent experiment with 5 mice per group. **G-H** data represents 2 independent experiments with 2-3 mice per group. $*P<0.05$, $**P<0.01$, $***P<0.005$, unpaired t-test.
