# Supplementary material for: Human Metapneumovirus Reinfection in Aged Mice Recapitulates Increased Disease Severity in Elderly Humans Infected with Human Metapneumovirus
Source: Immunohorizons. Author manuscript; Available in PMC 2023 Jul 5. (PMC10321313; doi:10.4049/immunohorizons.2300026)
Supplement: Supplementary file 1 [file NIHMS1908191-supplement-Supplemental_1.pdf]

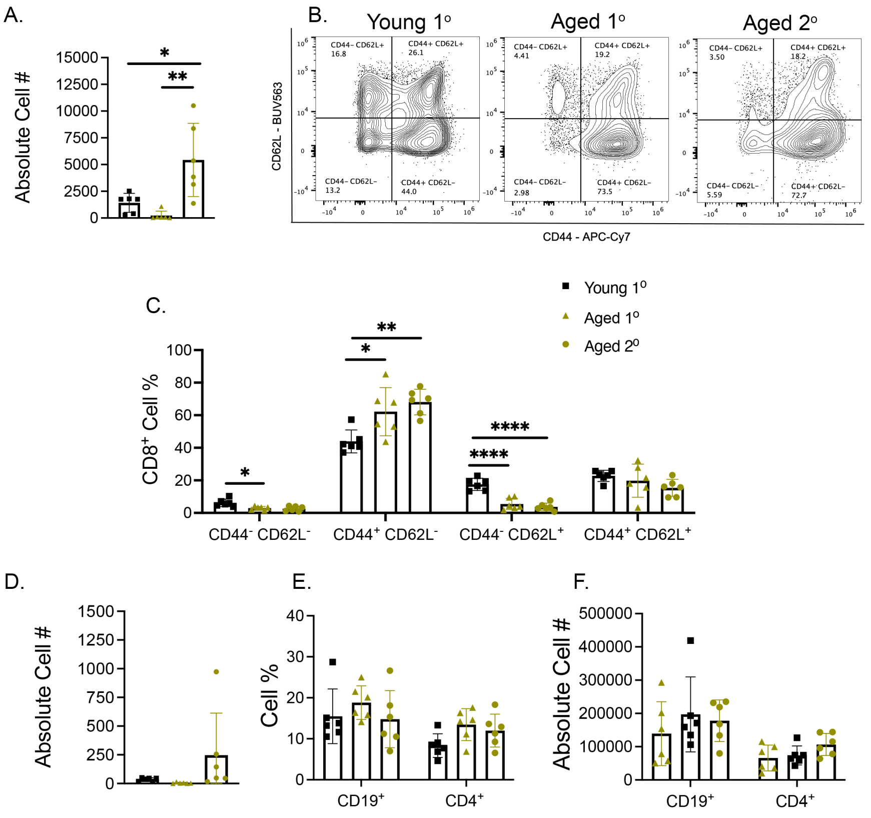

Supplemental Figure 1. Aged re-challenged mice have fewer naive CD8$^+$ CD44$^-$ CD62L$^+$ T cells but no difference in CD19$^+$ or CD4$^+$ T cells.

(A) Aged re-challenged mice had increased absolute cell number of tet$^+$ CD8$^+$ T cells. **(C)** Representative flow plots showing CD44 and CD62L expression on CD8$^+$ T cells. **(C)** Aged re-challenged mice had fewer CD8$^+$ CD44$^-$ CD62L$^+$ T cells and increased CD8$^+$ T cells expressing CD44$^+$ CD62L$^-$. **(D)** Aged re-challenged had a modest increase in absolute cell number of tet$^+$ CD8$^+$ memory T cells. **(E-F)** There was no difference in cell percent or absolute cell number of CD19$^+$ or CD4$^+$ cells between the groups. Data represent 3 independent experiments with 5-6 mice per experiment. *P<0.05, **P<0.01, ****P<0.0001, one-way ANOVA.
