# Supplementary material for: Human Metapneumovirus Reinfection in Aged Mice Recapitulates Increased Disease Severity in Elderly Humans Infected with Human Metapneumovirus
Source: Immunohorizons. Author manuscript; Available in PMC 2023 Jul 5. (PMC10321313; doi:10.4049/immunohorizons.2300026)
Supplement: Supplementary file 2 [file NIHMS1908191-supplement-Supplemental_2.pdf]

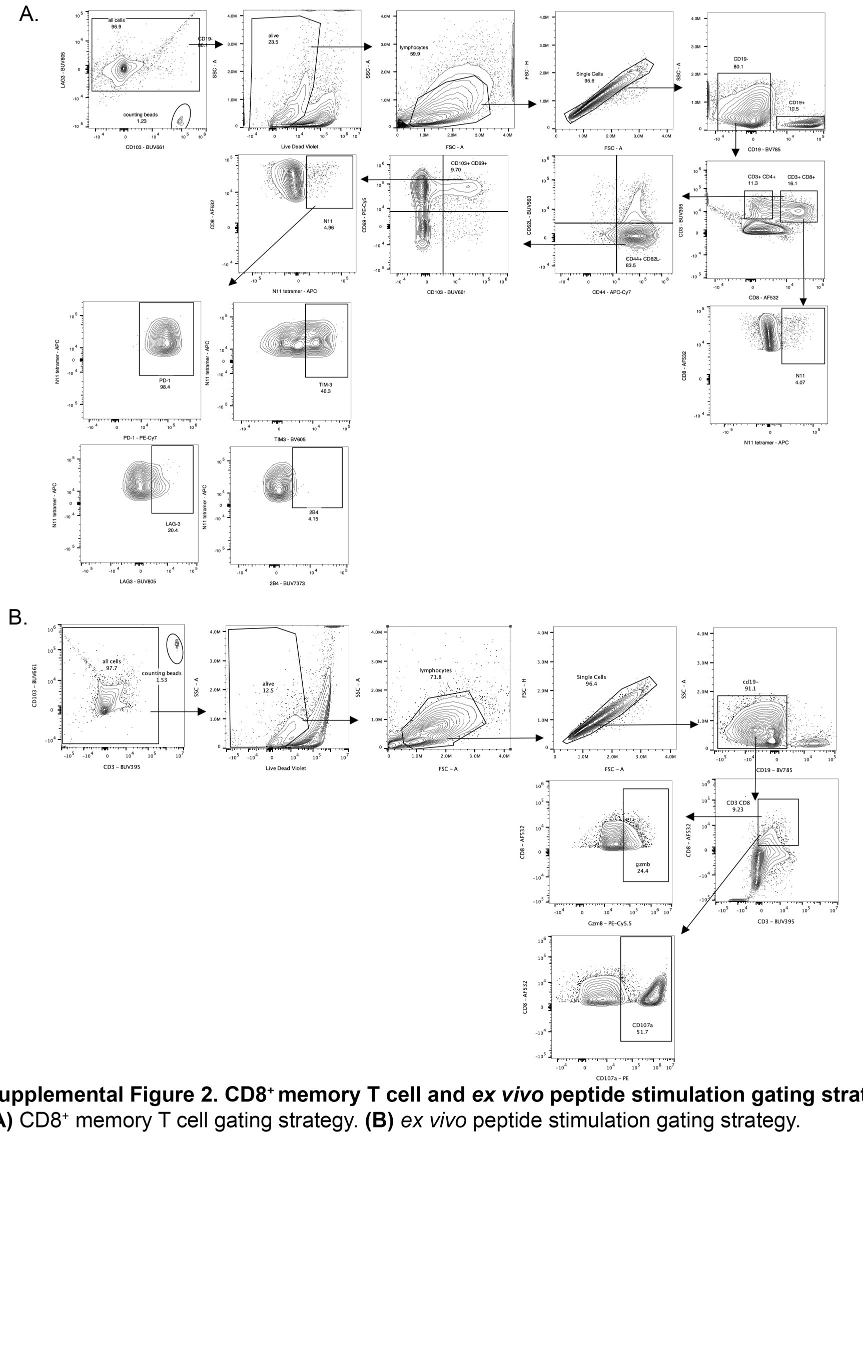

Supplemental Figure 2. CD8⁺ memory T cell and *ex vivo* peptide stimulation gating strategies. **(A)** CD8⁺ memory T cell gating strategy. **(B)** *ex vivo* peptide stimulation gating strategy.
