# Supplementary material for: Human Metapneumovirus Reinfection in Aged Mice Recapitulates Increased Disease Severity in Elderly Humans Infected with Human Metapneumovirus
Source: Immunohorizons. Author manuscript; Available in PMC 2023 Jul 5. (PMC10321313; doi:10.4049/immunohorizons.2300026)
Supplement: Supplementary file 3 [file NIHMS1908191-supplement-Supplemental_3.pdf]

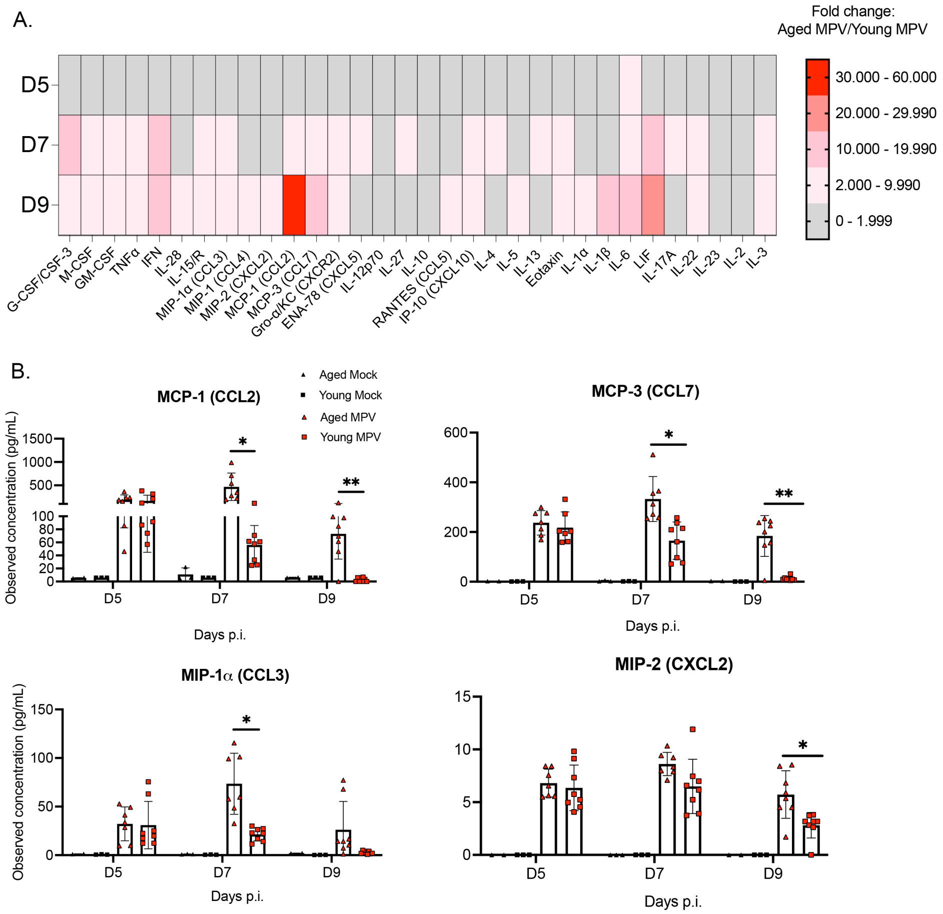

Supplemental Figure 3. Aged mice had increased T cell chemoattractants in lung late during HMPV infection.

(A) Fold change aged MPV/young MPV heat map of chemokine and cytokine expression from lung homogenates. **(B)** Raw data from select chemokines.

*P<0.05, **P<0.01, Two-way ANOVA.
