# Supplementary material for: Human Metapneumovirus Reinfection in Aged Mice Recapitulates Increased Disease Severity in Elderly Humans Infected with Human Metapneumovirus
Source: Immunohorizons. Author manuscript; Available in PMC 2023 Jul 5. (PMC10321313; doi:10.4049/immunohorizons.2300026)
Supplement: Supplementary file 4 [file NIHMS1908191-supplement-Supplemental_4.pdf]

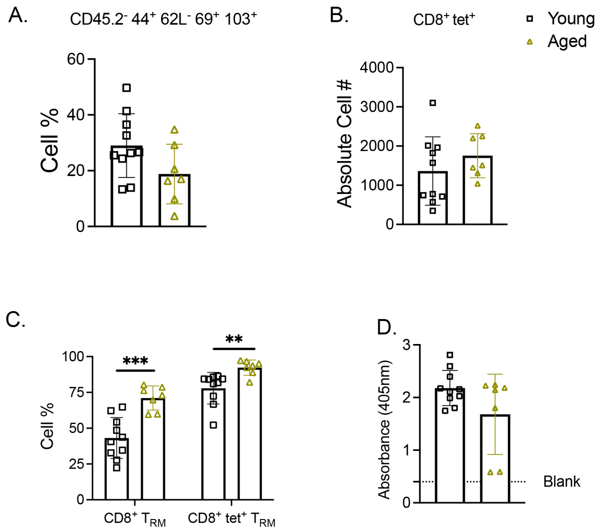

Supplemental Figure 4. Aged mice had increased PD-1 expression on CD45.2$^-$ CD8$^+$ T cells in the lung 40 days p.i.
(A) There was no difference between age groups in cell percent of CD45.2$^-$ CD44$^+$ CD62L$^-$ CD69$^+$ CD103$^+$ CD8$^+$ TRM cells. **(B)** There was also no difference in CD8$^+$ tet$^+$ absolute cell number. **(C)** Aged bulk CD8$^+$ and CD8$^+$ tet$^+$ TRMs had increased cell percent PD-1 expression. **(D)** There was no difference in HMPV IgG production as measured by HMPV ELISA. Data represent 2 independent experiments with 3-5 mice per experiment. **P<0.01,***P<0.005, unpaired t-test.
