# Supplementary material for: Human Metapneumovirus Reinfection in Aged Mice Recapitulates Increased Disease Severity in Elderly Humans Infected with Human Metapneumovirus
Source: Immunohorizons. Author manuscript; Available in PMC 2023 Jul 5. (PMC10321313; doi:10.4049/immunohorizons.2300026)
Supplement: Supplementary file 5 [file NIHMS1908191-supplement-Supplemental_5.pdf]

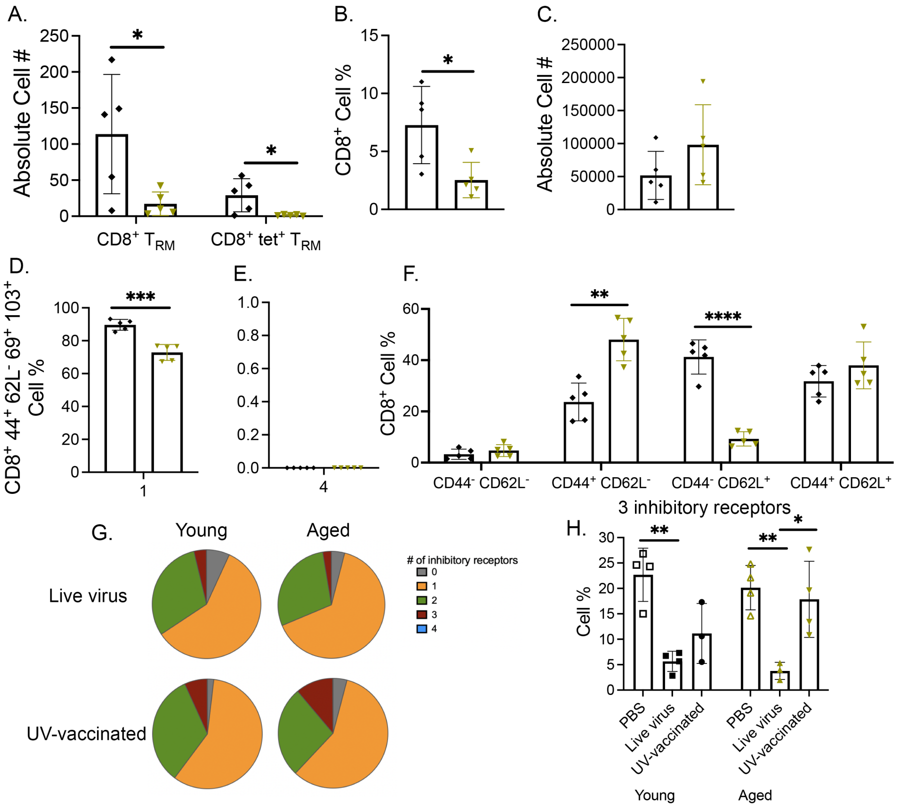

Supplemental Figure 5. Aged mice re-stimulated with HMPV cognate peptide adjuvanted with LPS or vaccinated with UV-inactivated virus had impaired CD8⁺ memory T cell response.
(A) Aged mice accumulated fewer bulk and tet⁺ CD8⁺ TRMs. **(B)** Aged mice also produced fewer tet⁺ CD8⁺ T cells compared to young mice. **(C)** There was no difference in CD8⁺ absolute cell number between the age groups. **(D)** Young mice had increased expression of only one inhibitory receptor on CD8⁺ TRMs. **(E)** Neither age group expressed all four inhibitory receptors (i.e. PD-1, TIM-3, LAG-3, and 2B4) on CD8⁺ TRMs. **(F)** Aged mice had increased CD8⁺ CD44⁺ CD62L⁻ T cells but had a smaller pool of naive CD44⁻ CD62L⁺ CD8⁺ T cells compared to young mice. (G) SPICE pie charts of combinatorial analysis of Boolean gating of 0, 1, 2, 3, or 4 inhibitory receptor expression. Inhibitory receptors assessed: PD-1, TIM-3, LAG-3, 2B4. Aged and young vaccinated mice had increased co-expression of 3 inhibitory receptors. **A-F** data represents 1 independent experiment with 5 mice per group. **G-H** data represents 2 independent experiments with 2-3 mice per group. *P<0.05, **P<0.01, ***P<0.005, unpaired t-test.
