# Supplementary material for: Human Metapneumovirus Reinfection in Aged Mice Recapitulates Increased Disease Severity in Elderly Humans Infected with Human Metapneumovirus
Source: Immunohorizons. Author manuscript; Available in PMC 2023 Jul 5. (PMC10321313; doi:10.4049/immunohorizons.2300026)
Supplement: Supplementary file 6 [file NIHMS1908191-supplement-Supplemental_6.pdf]

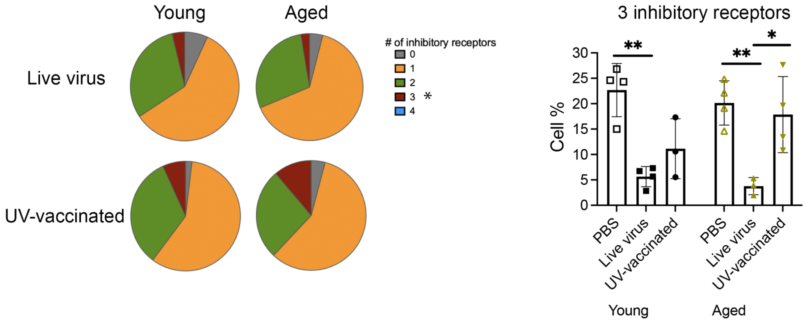

Supplemental Figure 6. Aged and young vaccinated mice had signficiantly increased co-expression of 3 inhibitory receptors.
SPICE pie charts of combinatorial analysis of Boolean gating of 0, 1, 2, 3, or 4 inhibitory receptor expression. Inhibitory receptors assessed: PD-1, TIM-3, LAG-3, and 2B4. Aged and young vaccinated mice had increased co-expression of 3 inhibitory receptors with no significant differences in 0, 1, 2, or 4 inhibitory co-expression. Data represents 2 independent experiments with 2-3 mice per group *P<0.05, **P<0.01, one-way ANOVA.
